# Multiple I-Type Lysozymes in the Hydrothermal Vent Mussel *Bathymodiolus azoricus* and Their Role in Symbiotic Plasticity

**DOI:** 10.1371/journal.pone.0148988

**Published:** 2016-02-16

**Authors:** Camille Detree, Apolline Chabenat, François H. Lallier, Nori Satoh, Eiichi Shoguchi, Arnaud Tanguy, Jean Mary

**Affiliations:** 1 Sorbonne Universités, UPMC Univ Paris 06, CNRS UMR 7144, Adaptation et Diversité en Milieu Marin, Equipe ABICE, Station Biologique de Roscoff, 29680 Roscoff, France; 2 Marine Genomics Unit, Okinawa Institute of Science and Technology, Onna, Japan; National Research Laboratory of Defense Proteins, REPUBLIC OF KOREA

## Abstract

The aim of this study was first to identify lysozymes paralogs in the deep sea mussel *Bathymodiolus azoricus* then to measure their relative expression or activity in different tissue or conditions. *B*. *azoricus* is a bivalve that lives close to hydrothermal chimney in the Mid-Atlantic Ridge (MAR). They harbour in specialized gill cells two types of endosymbiont (gram—bacteria): sulphide oxidizing bacteria (SOX) and methanotrophic bacteria (MOX). This association is thought to be ruled by specific mechanism or actors of regulation to deal with the presence of symbiont but these mechanisms are still poorly understood. Here, we focused on the implication of lysozyme, a bactericidal enzyme, in this endosymbiosis. The relative expression of Ba-lysozymes paralogs and the global anti-microbial activity, were measured in natural population (Lucky Strike -1700m, Mid-Atlantic Ridge), and in *in situ* experimental conditions. *B*. *azoricus* individuals were moved away from the hydrothermal fluid to induce a loss of symbiont. Then after 6 days some mussels were brought back to the mussel bed to induce a re-acquisition of symbiotic bacteria. Results show the presence of 6 paralogs in *B*. *azoricus*. In absence of symbionts, 3 paralogs are up-regulated while others are not differentially expressed. Moreover the global activity of lysozyme is increasing with the loss of symbiont. All together these results suggest that lysozyme may play a crucial role in symbiont regulation.

## Introduction

*Bathymodiolus azoricus* is a bivalve mollusc of the Mytilidae family that lives around hydrothermal vents on the Mid-Atlantic Ridge (MAR) [1]. This mussel hosts two types of endosymbiotic γ-proteobacteria, one sulphide-oxidizing (SOX) and one methanotrophic (MOX), in specialized gill cells called bacteriocytes [2–5]. They provide the host with carbon and energy from the oxidation of reduced compound present in the environment (H_2_S, CH_4,_ H_2_). This dual endosymbiosis is characterized by a strong plasticity [6] inducing a change in SOX/MOX ratio when the concentration of methane or sulphide changes [7–10] or a loss of symbionts in the absence of reduced compounds [11]. The metabolism of these symbiotic bacteria is relatively well understood compared with that of the host [12–15]. *B*. *azoricus* is indeed a mixotrophic organism maintaining its heterotrophic filtration capacity together with its chemotrophic symbioses [16]-[17]. Fiala-Médioni and co-authors showed in a congeneric species (*B*. *thermophilus*, Galapagos Rift), that the assimilation of nutrients from symbionts to host, is largely based on the intracellular digestion of bacteria using the lysosome/lysozyme system [18]-[19]. More recently, Broderick [20] made the general hypothesis that, in metazoans, digestion and immunity could have a common origin. Indeed, many proteins such as proteases, chitinases, antimicrobial peptides or lysozymes are implicated in both processes. In addition, immunity is thought to be crucial in symbiotic associations to recognize, maintain or regulate symbionts populations [21–23].

Lysozymes (EC 3.2.1.17) are described as anti-bacterial proteins that catalyse the hydrolysis of the β-1,4-glycosidic bonds of heteropolymers of N-acetylmuramic acid and N-acetylglucosamine in bacterial cell walls [24]-[25] and seems to have a role in both immunity and digestion [26–28]. They are divided in six types called goose type (LysG or g-type), chicken type (LysC or C-type), invertebrate type (LysI or i-type), phage-type, bacterial-type and plant-type lysozymes according to differences in their structure and biological functions [29]. I-type lysozymes have only been reported in invertebrates phyla including Annelida [30], Nematoda [31], Arthropoda [32], Echinodermata [33], and Mollusca [34]. Many of the studies recently conducted on lysozymes in mollusk species focused on their role in immunity but none of them raised the question of their involvement in the management of symbiotic associations. In other symbiotic models such as aphids/*Buchnera*, high levels of lysozyme expression have been reported in bacteriocytes raising the question of the role of lysozymes in the breakdown of the symbiosis and/or the elimination of other microbial intruders [35]. Within the general frame of our researches on the biology of *B*. *azoricus*, the main goal of this study was to provide a comprehensive knowledge on the implication of lysozymes in the regulation of the symbiotic association between *B*. *azoricus* and SOX/MOX bacteria. In this study, we conducted an original *in situ* experiment at Lucky Strike vent site (1700 m depth), consisting in the translocation of mussels away from their native mussel bed to examine the kinetics of symbiont loss, followed after a short period by a return to their original mussel bed to test the possibility of symbiont re-acquisition. Following a characterisation of the different paralogs of lysozymes found in *B*. *azoricus* transcriptome, we measured their relative expression in different tissues of mussels from natural population and in gills of individuals from *in situ* experiments in relation with their symbiont content.

## Materials and Methods

### Animal collection: field sample and *in situ* experiments

Deep sea mussels *Bathymodiolus azoricus* were collected from one hydrothermal vent site (Lucky Strike, population of Montsegur, 37°17,286’ N, 32°16,530’ W; 1700 m depth) located on the Mid Atlantic Ridge during the BioBaz and MoMARSAT cruises (July/August 2013). The sampling was done using the Remote Operating Vehicle (ROV) Victor 6000 on the Research Vessel “Pourquoi pas?”. Around 30 mussels representing the natural population were collected and placed in hermetic boxes then brought on board within 2 hours. Gills, mantle and digestive gland were immediately dissected, frozen in liquid nitrogen, and stored at -80°C until further analysis.

For *in situ* experiments, between 15 and 30 mussels were put in several wire cages and 5 of them were placed a dozen meters away from the hydrothermal venting area. This condition was called “basalt”. Cages were brought back on board after 6 days (BT1), 20 days (BT2) and 27 days (BT3) on basalt. In addition, after 6 days on basalt (T1), 2 cages were translocated back from basalt to their original mussel bed in the venting area, and brought back to the research vessel after respectively 14 days (TR1.2) and 21 days (TR1.3) on mussel bed. Immediately after collection, gills were dissected, frozen in liquid nitrogen and stored at -80°C until further analysis.

### Quantification of symbiont content by real-time PCR

Genomic DNA of both mussels and symbiont bacteria was extracted from gill tissue using CTAB extraction procedure (2% CTAB, 1% PVP, 1.4 M NaCl, 0.2%^®^ mercaptoethanol, 100 mM Tris HCl pH8, 0.1 mg. mL^-1^ proteinase K, 1 mg.mL^-1^ egg-white lysozyme). Tissues were incubated for 2 h at 60°C for complete digestion. An equal volume of chloroform-isoamyl alcohol (24:1) was then added to the solution and tubes were inverted gently to mix for 5 min. The mix was centrifuged 10 min at 12,000 rpm at 4°C and the supernatant was carefully collected into a new tube, then DNA was precipitated with 2/3 volume of cold isopropanol (1 h at -20°C). The DNA pellet was recovered by centrifugation (12,000 rpm/4°C/20 min), washed with 70% cold ethanol, air-dried and re-suspended in 200 μL RNase free sterile water. Quantity and quality of DNA were assessed *via* UV absorbance (OD260/280/230), using a Nanodrop ND-1000 spectrophotometer (Nanodrop Technologies, Delaware, USA). The relative quantity of symbionts was estimated by real-time PCR amplification according to the protocol available in Boutet et al. (2011). All experiments were carried out using the 2× Lightcycler^®^480 SYBR Green I Master mix (Roche Diagnostics, Mannheim Germany), 70 nM of each primer, 2.1 μL of diluted DNA (2.5 ng) in a final volume of 5 μL and PCR were run in Light Cycler 480 machine (Roche Diagnostics, Mannheim Germany). The amplification was carried out in triplicate as follows: initial enzyme activation at 94°C for 15 min followed by 45 cycles of 94°C for 15 sec, 60°C for 30 sec and 72°C for 30 sec. A 120 bp-fragment of cytosolic malate dehydrogenase gene (MDH) from the host was used as an internal PCR control (Table 1). The relative quantity of SOX and MOX symbiont was estimated by using the comparative Ct method formula: Relative quantification = 2^-ΔCt^, (ΔCt = Ct_16S_-Ct_MDH_).

**Table 1:** Primers used in qPCR.

Genes	Primers sequence 5’-3’
Cytosolic malate dehydrogenase (host) (MDH)	For: 5’‐ ATGGAGGAAAGAGATATGGCACTGAGCGT‐3’
	Rev: 5’‐TAACATTAAACATAGCCTAGGAACCTAATG‐3’
Methanotrophic symbiont 16S (MOX)	For 5’‐GTGCCAGCMGCCGCGGTAA‐3’
	Rev 5’‐GCTCCGCCACTAAGCCTATAAATAGACC‐3’
Sulphide-oxidizing symbiont 16S (SOX)	For 5’‐GAGTAACGCGTAGGAATCTGC‐3’
	Rev:5’‐CGAAGGTCCTCCACTTTACTCCATAGAG‐3’
Ribosomal protein L15 (RpL15)	For 5’-TATGGTAAACCTAAGACACAAGGAGT-3’
	Rev 5’-TGGAATGGATCAATCAAAATGATTTC-3’
Ba-lysozyme 1	For 5’- ATGTCTCCTCGAGTTTATTTGGTGTTACT-3’
	Rev 5’- CAACCTGACAGATACAGCTATACAT-3’
Ba-lysozyme 2	For 5’- TGTGCTAAGGACTATGAATGTTCCAA-3’
	For 5’- TCCCAATAACCAAGAGTTGTTGGTTT-3’
Ba-lysozyme 3	For 5’- TGTCAGGTTGAATCGCACTGCCACCCCAT-3’
	Rev 5’-GGATTTGTACACCCTGCTGGTCCTCCGTT-3’
Ba-lysozyme 4	For 5’- ATGAAAAAGATGATGATTATTGCAGGAAT-3’
	Rev 5’- GATTGCACCCATGATGATGACGCGAGTGTG-3’
Ba-lysozyme 5	For 5’- TGTCTTCAGTGTATCTGTGACGCGGAGAC-3’
	Rev 5’- TTAGCACACTCCTCAAAACTGTTTCCTGG-3’
Ba-lysozyme 6	Rev 5’- GAGACTGTGACTAGAACAGCCAACATTCCT-3’
	For 5’-TACGCTAGAATGCACAACGGTGGACC-3’

### Lysozymes characterisation: sequences, structure and phylogeny

All *B*. *azoricus* lysozymes sequences used in this study have been obtained from previous existing transcriptome database. Briefly, RNA was extracted from various tissues (gill, mantle, and digestive gland) of *B*. *azoricus* samples collected in Menez Gwen site (37°50’ N, 31°31’ W, 850 m depth) during a previous cruise (BIOBAZ initiale in 2011). Sequencing was performed at Marine Genomics Unit of Okinawa Institute of Science and Technology (OIST-Japan) using the standard Illumina RNA library preparation protocol and a single lane of the HiSeq 36 bases pair-end approach. Contigs were *de novo* assembled with Velvet-Oases version 0.2.08 [36] using the Bioinformatic platform of Roscoff. Optimal kmer length was determined by assembling one genotype at all possible kmer values from 20 to 60 using Velvet version 0.7.55 [37]. The kmer length of 51 was selected for further assemblies. The Velvet assembly generated 62987 contigs and all consensus transcript sequences were searched against protein databases including NCBI non-redundant (nr) database and Swiss-Prot using BLASTx with a cutoff e-value of e-03. BLAST2GO was used to assign gene ontology (GO) annotation [38]. Illumina sequences generated in this study have been deposited in Data Bank of Japan (DDBJ) Sequence Read Archive (DRA, http://trace.ddbj.nig.ac.jp/dra/) with accession number DRA 004082.

The amino acid sequences of Ba-lysozymes were determined using the Expert Protein Analysis System (http://www.expasy.org/tanslate/). Multiple alignments were conducted with the ClustalW program (http://npsa-prabi.ibcp.fr/). Signal peptides were predicted by SignalP4.1 server (http//www.cbs.dtu.dk/services/SignalP/). The three-dimensional model of Ba-lysozyme 4, 5 and 6 were constructed by comparative modelling with Modeller 9v13 program. The structure of the i-type lysozyme isolated from the common orient clam *Metrix lusaria*, crystallized with 3 N-Acetyl-D-Glucosamine (NAG) as substrates (pdb entry: 3AB6), was used as template. One hundred models were generated for each Ba-lysozyme paralog and their quality assessed using the Modeller Objective Function parameter. The reliability of modelled structures was validated by Ramachandran plot analysis using PROCHECK (http://www.ebi.ac.uk/thornton-srv/databases/pdbsum/Generate.html). A phylogenetic tree of bivalve lysozymes, based on the alignment of 68 residues within the lysozyme domain, was constructed with Seaview 4.5.4 software using the neighbor-joining (BioNJ) method with 1000 bootstrap resampling of the data set.

#### Lyzozymes expressions: RNA extraction and qPCR

The mRNA expression of lysozymes paralogs was analysed by real-time PCR. Total RNA was extracted from mussel gills, digestive gland and mantle by using Tri-Reagent (Sigma) according to the manufacturer's instructions. Both quantity and quality of RNA were assessed *via* UV absorbance (OD260/280/230) using a Nanodrop ND-1000 spectrophotometer (Nanodrop Technologies, Delaware, USA). Two μg of total RNA were reverse transcripted using M-MLV reverse transcriptase (Promega), an anchor-oligo(dT) primer (5′-CGCTCTAGAACTAGTGGATCT-3′) and random hexamers (Promega). A volume of 2 μL of each diluted reverse transcription product (1:200) was subjected to real-time PCR in a final volume of 5 μL containing 40 nM of each specific primer and 2×Lightcycler^®^480 SYBR Green I Master mix (Roche Diagnostics, Mannheim Germany). The amplification was carried out as follows: initial enzyme activation at 95°C for 15 min, then 45 cycles of 95°C for 10 sec and 60°C for 30 sec. A dissociation curve was generated and PCR efficiency was estimated for each primer pair. All primer pairs tested generated a single peak in the dissociation curve and a PCR efficiency of 95 to 100%. Relative expression of each gene (fold-change) was calculated according to comparative Ct method using the formula: Ratio = 2^-ΔΔCt^ (with ΔCt_geneX_ = Ct_GeneX_-Ct_RiboL15_; ΔΔCt = ΔCt_geneX_-meanΔCt_geneX_). This formula was chosen to standardize the levels of gene expression to a mean of zero and consequently give the same weight to all response variables. A fragment of ribosomal protein L15 gene (RpL15) from the host was used as the internal PCR control (Table 1). We validated the RpL15 after having observed very low (less than 5%) variation of its expression in all samples (both experimental and field individuals) for gill tissue.

#### Lysozyme activity

Lysozyme activity was determined according to the method of Shugar [39]. Briefly, frozen gills were crushed in 150 μL of PBS at room temperature (0.1 M, pH7.2) followed by sonication and centrifugation (5,000 g/15 min/4°C). The supernatant containing proteins was kept and pellet was discarded. The protein concentration was determined using the Bio-Rad Protein Assay kit (Bio-Rad, Marnes-la-Coquette, France). A suspension of *Micrococcus lysodeikticus* (2.5 mg/mL) was prepared in PBS (0.1M, pH7.2). The assay was done in a 96-well microplate where 30 μL of sample was added to 170 μL of bacterial suspension. After 10 minutes, absorbance at 600 nm was recorded at 20°C on the Xenius Safas^™^ spectrophotometer. Egg-white lysozyme was used for standard curve. All measurements of lysozyme activity were done in triplicate.

### Statistical analysis

Data from quantitative real-time PCR experiments were analysed with a Kruskal-Wallis test GraphPad Prism,USA (www.graphpad.com*)* followed by a Dunn’s Multicomparison Test. For tissue specificity, an One way analysis of variance test was performed followed by a Turkey post test. Differences were considered statistically significant when P≤0.05.

## Results and Discussion

### Characterisation and phylogenetic position of Ba-lysozymes among bivalve homologs

**Fig 1:**
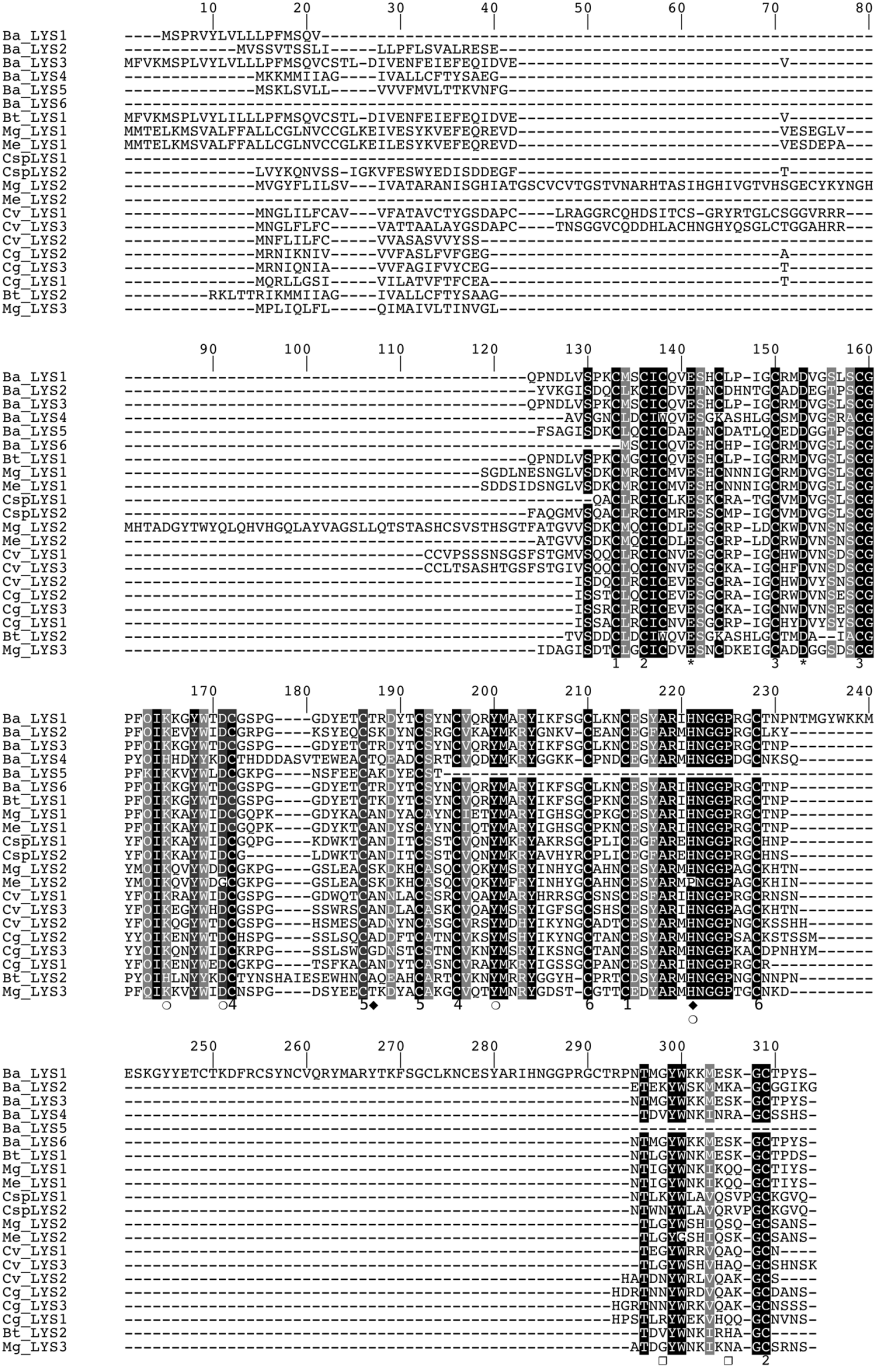
Multiple alignment of Ba-lysozyme paralogs with other i-type lysozymes from bivalve molluscs. The black and grey regions indicate positions where amino acid residues are conserved or highly similar, respectively. The asterisks indicate the two catalytic residues (141 & 153). Amino acid residues contributing to substrate binding are indicated by empty circles, those contributing to destabilase activity are indicated by black diamonds and those contributing to a potential dimerization are indicated by empty squares. Disulphide bridges and cysteine residues implicated are numbered from 1 to 6.

The analysis of the EST database of *Bathymodiolus azoricus* revealed the existence of six paralogs of lysozymes, referred Ba-lysozymes 1 to 6. With the exception of Ba-lysozyme 5 that is incomplete in its C-terminal region, all Ba-lysozymes identified are full-length, and contain 111 to 201 residues (Fig 1, Table 2, Figure A in S1 File) with a p*I* ranging from 5.80 to 8.96.

**Table 2:** Molecular properties of the six lysozymes paralogs of *Bathymodiolus azoricus*.

Name	Length	Signal peptide	MW (kDa)*	Theoretical pI*	Cys residues	Disulfide bonds **
Ba-Lysozyme 1	201	1–19	22.9/20.7	9.01/8.96	20	6
Ba-Lysozyme 2	144	1–20	15.9/13.8	7.88/7.60	14	6
Ba-Lysozyme 3	163	1–24	18.4/15.7	6.10/5.67	15/14	6
Ba-Lysozyme 4	143	1–22	15.7/13.4	5.8/5.57	13/12	6
Ba-Lysozyme 5	88	1–21	9.5/7.2	4.55/4.24	9	3
Ba-Lysozyme 6	111	No	12.5	8.56	13	5

*Molecular weight and p*I* were determined with or without the signal peptide

** Number of predicted disulphide bridges from the structural 3D model

Cys residues: Number of cysteine residues

**Fig 2:**
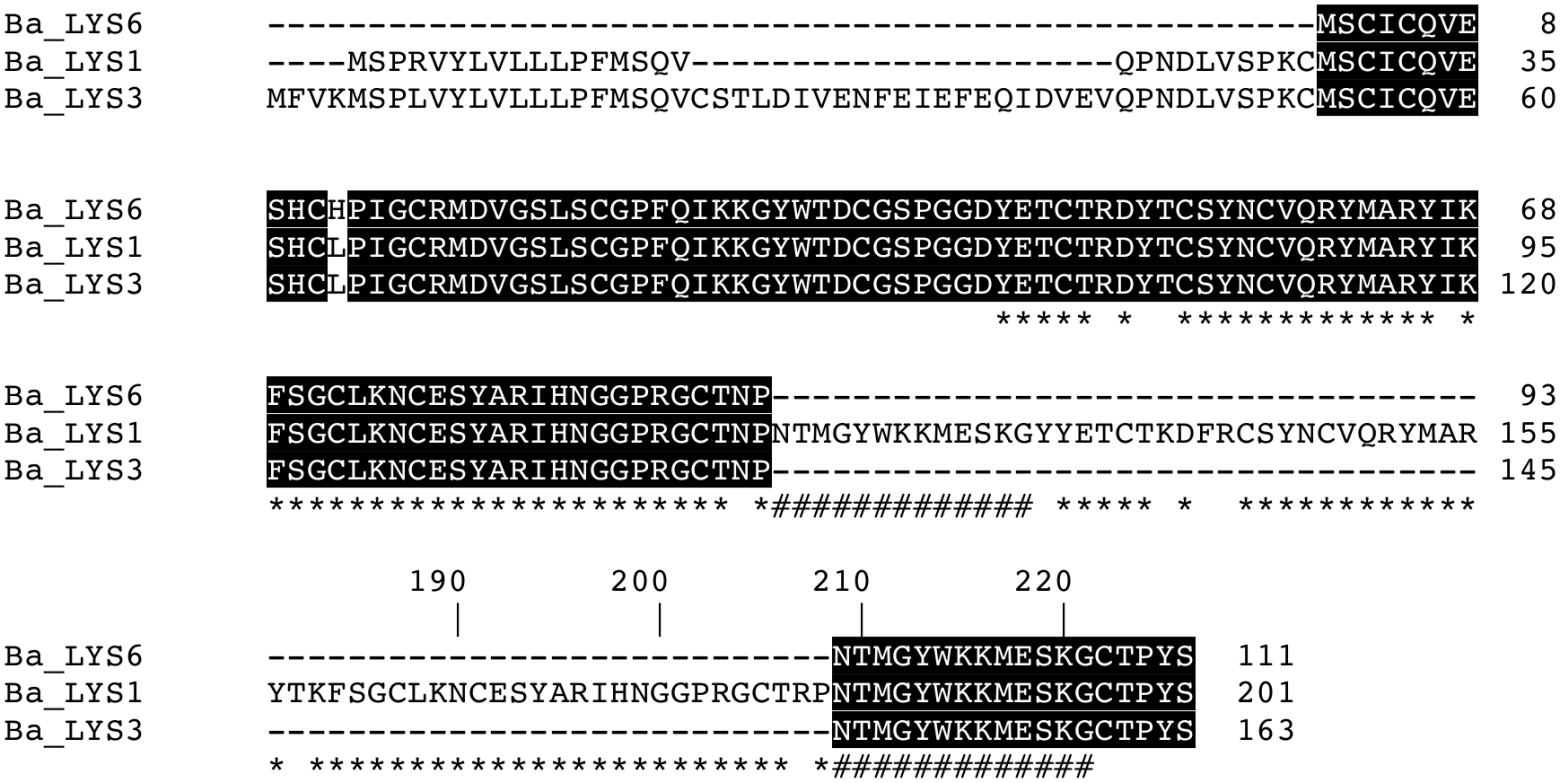
Amino acid alignment of the Ba-lysozyme 1, 3, 6. The sequences indicated by # and * are those which are repeated in the internal core sequence of Ba-lysozyme1.

**Fig 3:**
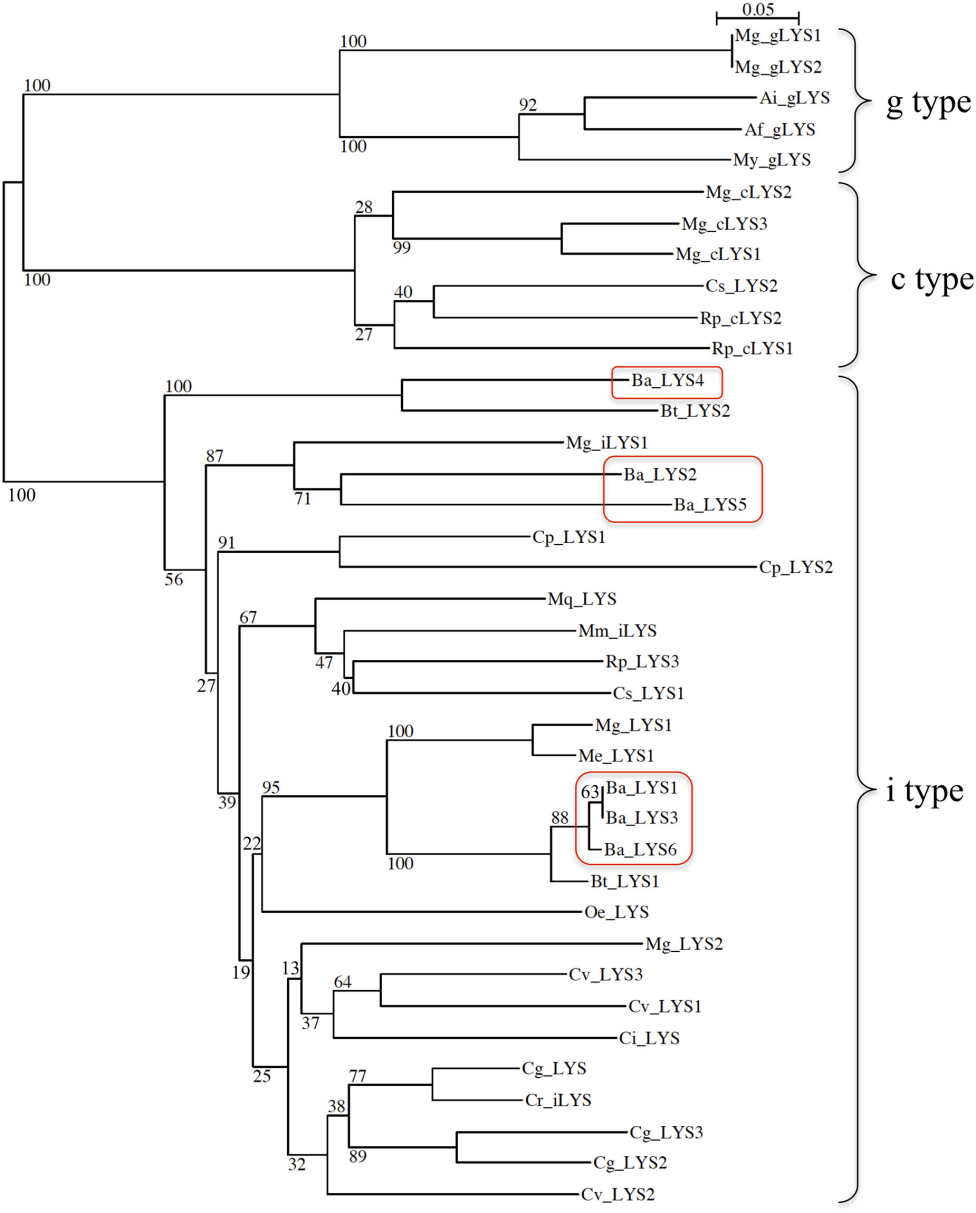
Phylogenetic analysis of lysozymes among bivalve. The distance tree was obtained by neighbor joining with 1000 bootstrap resampling of the data set (value below the branches, Seaview 4.5.4). Red boxes indicate the *Bathymodiolus azoricus* paralogus lysozymes. The UniProt accession no. and the species are as follows: AF334665 (*Mytillus galloprovincialis*, Mg_LYS1), AB298451 (*Mytillus* Ai_gLYS (*Argopecten irradians*, Q2HNY7_ARGIR), Af_gLYS (*Azumapecten farreri*, Q075V3_9BIVA), Bt_LYS1 (*Bathymodiolus thermophilus*, Q8ITU0_BATTH), Ci_LYS (*Chlamys islandica*, Q9BI29_CHLIS), Cg_LYS (*Crassostrea gigas*, LYS_CRAGI), Cg_LYS2 (*Crassostrea gigas*, A3KDX2_CRAGI), Cg_LYS3 (*Crassostrea gigas*, A9CPZ8_CRAGI), Cp_LYS1 (I6ZA43_CRIPL, *Cristaria plicata*), Cp_LYS2 (*Cristaria plicata*, I6ZIX7_CRIPL), Cr_iLYS (*Crassostrea rivularis*, F1KBQ5_9BIVA), Cs_LYS1 (*Cyclina sinensis*, G9BNP4_CYCSN), Cs_LYS2 (*Cyclina sinensis*, F6K6C5_CYCSN), Cv_LYS1 (*Crassostrea virginica*, LYS1_CRAVI), Cv_LYS2 (*Crassostrea virginica*, LYS2_CRAVI), Cv_LYS3 (*Crassostrea virginica*, LYS3_CRAVI), Me_LYS1 (*Mytilus edulis*, Q8ITU2_MYTED), Mg_iLYS1 (*Mytilus galloprovincialis*, A0A0C5PIW8_MYTGA), Mg_LYS1 (*Mytilus galloprovincialis*, Q8ITT9_MYTGA), Mg_LYS2 (*Mytilus galloprovincialis*, A5LHX1_MYTGA), Mg_cLYS1 (*Mytilus galloprovincialis*, I6QSK6_MYTGA), Mg_cLYS2 (*Mytilus galloprovincialis*, A0A0C5PIW0_MYTGA), Mg_gLYS1 (*Mytilus galloprovincialis*, J7II22_MYTGA), Mg_gLYS2 (*Mytilus galloprovincialis*, J7IAB9_MYTGA), Mg_cLYS3 (*Mytilus galloprovincialis*, A0A0C5PW39_MYTGA), Mm_iLYS (*Meretrix meretrix*, F6JX82_MERMT), Mq_LYS (*Mactra quadrangularis*, K9JF24_9BIVA), My_gLYS (*Mizuhopecten yessoensis*, H2EV82_MIZYE), Oe_LYS (*Ostrea edulis*, LYS_OSTED), Rp_cLYS1 (*Ruditapes philippinarum*, R9WZ60_RUDPH), Rp_cLYS2 (*Ruditapes philippinarum*, R9WXF4_RUDPH), Rp_LYS3 (*Ruditapes philippinarum*, C8CBP0_RUDPH).

This large range of p*I* was also observed in i-types lysozymes from the oyster *Crassostrea virginica* by Xue et al. [40] who suggested that the varying p*I* values could be linked to different functions. Indeed, in 2007 Xue et al. [28] stipulated that lysozymes that contain less protease-cutting sites and lower p*I* values could be associated with a digestive function as shown in other species [41,42]. In their mature form, the full-length Ba-lysozymes contain 12 to 20 cysteine residues, allowing the formation of 5 to 6 disulfide bridges, in accordance with the 3D structural models (Figure B in S1 File). Ba-lysozymes 1, 3 and 6 have a core of identical amino acid sequence except for one position (145 in Fig 1). However, Ba-lysozymes 1 and 3 have an N-terminal extension of 27 and 52 residues respectively, constituting part of a signal peptide, and share one motif (MSPR/LVYLVLLLPFMSQV). Moreover Ba-lysozyme 1 contains an additional internal sequence of 63 residues composed of the repetition of the flanking regions (Fig 2). With the exception of Ba-lysozyme 6, all the paralogs present a signal peptide (16 to 24 residues). For Ba-lysozyme 1, 2, 3, 4 and 6 the amino acids residues important for the lysosomal activity (substrate binding and catalysis) are conserved (Fig 1) [43] whereas for Ba-lysozyme 5, incomplete in its C-terminal part, two residues implicated in substrate binding are missing. Ba-lysozyme 2 presents two residues (Ser82 and His115) which, according to Kuwano et al. [43] could confer to this paralog a destabilase activity. Moreover, Ba-lysozyme 2 presents two lysines which may be involved in its dimerization, as was described for *Tapes japonica* i-type lysozymes [44]. All the paralogs shared a high similarity with lysozymes from others bivalves and shared the essential amino acids residues characteristic of i-type lysozymes. Furthermore, in a molecular phylogenetic analysis of bivalve lysozymes, the six Ba-lysozymes cluster with the different i-types (Fig 3). According to the tree, the paralogs Ba-lysozymes 1, 3 and 6 (Ba_LYS1, LYS3 and Ba_LYS6) are nearly identical and could represent allelic forms of the same gene whereas only one orthologous form has been detected in *Bathymodiolus thermophilus*. However, genotyping these three paralogs by quantitative PCR in several individuals indicated that they are all present within each individual (see below). This suggest that these three paralogs recently (after speciation) emerged by a duplication event. Moreover, the three paralogs also diverged by the length of the peptide signal (Fig 1) suggesting different functions.

Interestingly, we only identified i-type lysozymes in the hydrothermal mussel when other mussel species such as *M*. *galloprovincialis* exhibits the three i-, g- and c-type lysozymes [45]. C-type lysozymes remain less described in mollusk species with two in *M*. *galloprovincialis* and in the clam *Cyclina sinensis* [46], and one in the abalone *Haliotis discus hannai* [47]. Moreover c-type lysozymes have not be detected in the *Crassostrea gigas* genome and they are also absent from the phylum of Echinodermata and Urochordata [48]. G-type lysozymes have been identified in urochordate [49] and several mollusc species such as Zhikong scallop [50], Japanese scallop [51], the abalone [52], and mussel [53].

### Tissue specificity of Ba-lysozyme paralogs

**Fig 4:**
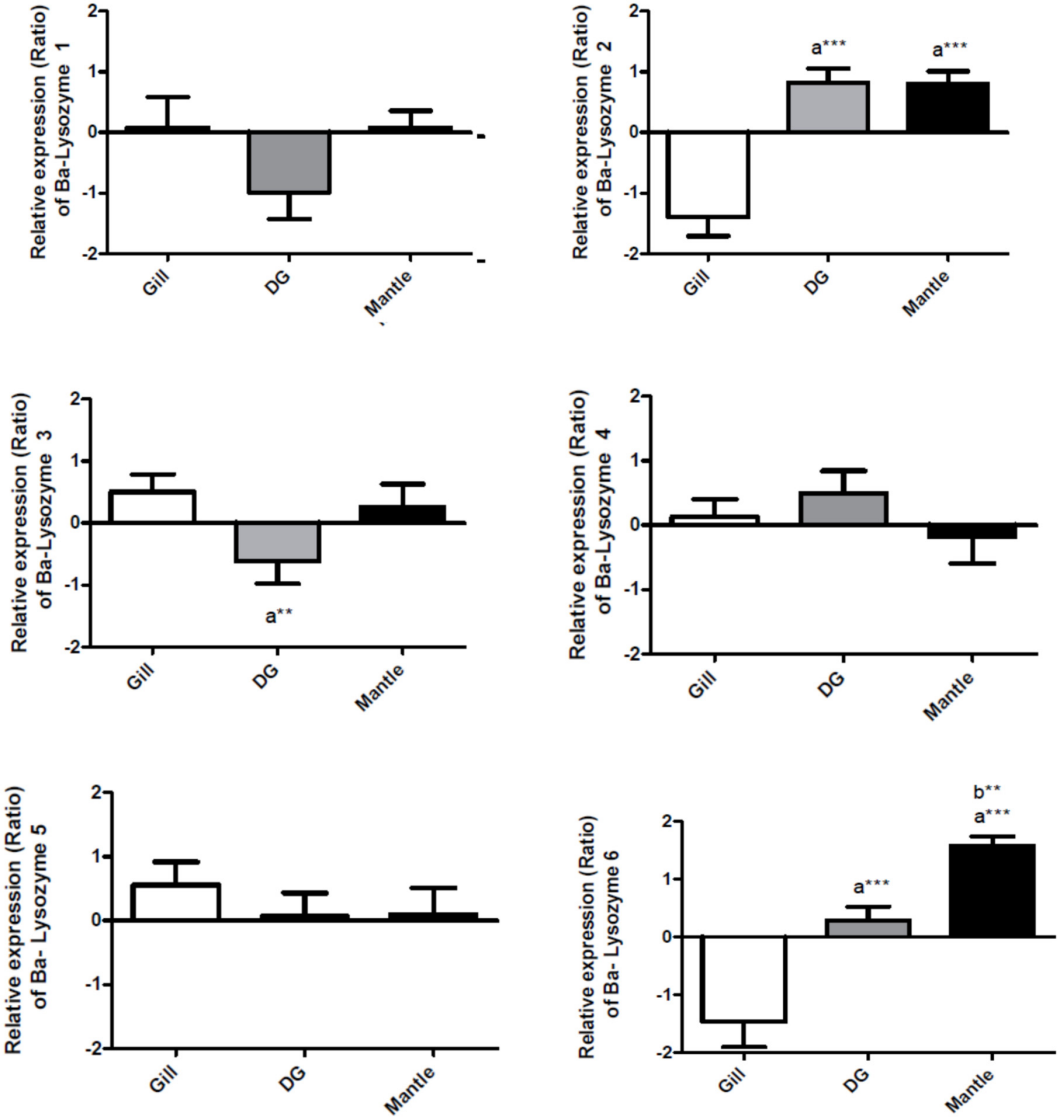
Tissue-specificity of different lysozymes paralogs expression. Bars show the relative expression level of mRNA in three tissues for each paralog according to the comparative Ct method formula: Ratio = 2^-ΔΔCt^ (with ΔCt_geneX_ = Ct_GeneX_-Ct_RpL15_; ΔΔCt = ΔCt_geneX_-meanΔCt_geneX_), with RpL15 used as an internal control. Gill (n = 31) in white, Digestive Gland (n = 30) in grey and Mantle (n = 32) in black. DG: Digestive gland. a: significantly different from Gill; b: Significantly different from DG (*: p<0.05, ** p<0.001, *** p<0.0001), (One-way analyse of variance followed by Turkey post test).

The tissue-specific expression pattern of the six *B*. *azoricus* lysozyme paralogs has been investigated by qPCR in two non-symbiotic tissues (digestive gland and mantle) and in the symbiotic tissue (gills) in mussels from the natural population (Lucky Strike). Three Ba-lysozyme paralogs showed a significant differential expression between tissues (Fig 4). Ba-lysozyme 3 appears to be up regulated in gills compared to digestive gland, whereas Ba-lysozyme 2 and Ba-lysozyme 6 are down regulated in gills compared to the two other tissues. In addition, Ba-lysozyme 2 exhibits a similar relative expression in both mantle and digestive gland, and Ba-lysozyme 3 expression is also similar in gills and mantle. Moreover, Ba-lysozyme 6 is significantly more expressed in the mantle than in digestive gland. Even if significant, ratios of expression between tissues remain relatively weak (no more than 3 fold). Interestingly, we observed a distinct tissue expression pattern between the Ba-lysozymes 1, 3 and 6 when these three sequences are almost identical with the exception of their signal peptide and large amino acid insertion in the Ba-lysozyme 1 (Fig 2). These amino acid differences might confer them different functions, which could explain their distinct tissue expression. The detection of a differential tissue expression pattern in the lysozyme family has been described in several other bivalve mollusc species: the pearl mussel *Hyriopsis cumingii* [54], the Pacific oyster *C*. *gigas* [55] and the eastern oyster *C*. *virginica* [56,57].

**Fig 5:**
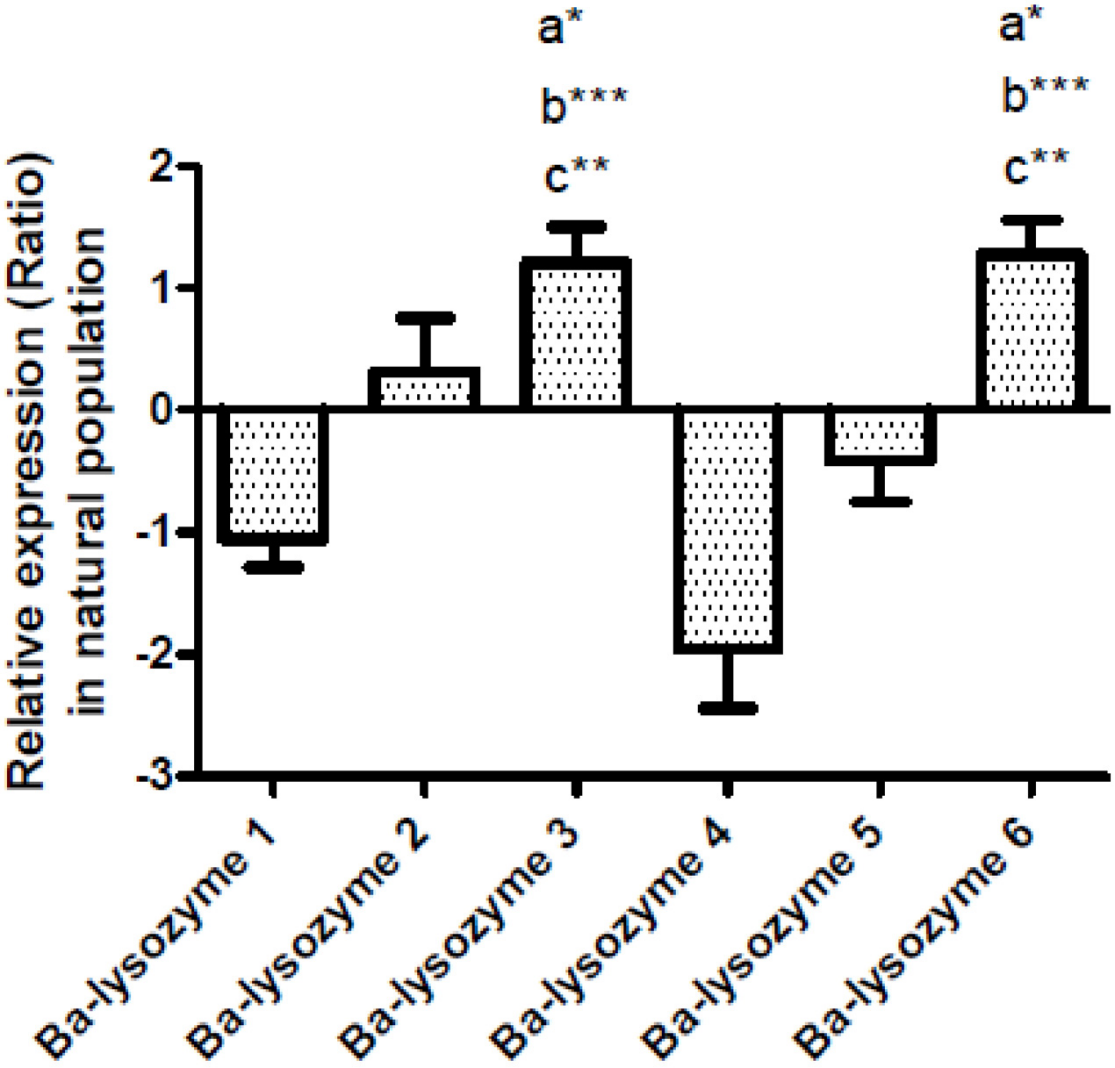
Relative expression level (Ratio) of the six i-type lysozymes in *B*.*azoricus* gills in natural population (n = 31). Bars show the relative expression level of mRNA for each paralog according to the comparative Ct method formula: Ratio = 2^-ΔΔCt^ (with ΔCt_geneX_ = Ct_GeneX_-Ct_RpL15_; ΔΔCt = ΔCt_geneX_-meanΔCt_geneX_), here RpL15 is used as an internal control. a: significantly different from Ba-lysozyme 1; b: significantly different from Ba-lysozyme 4 and c: significantly different from Ba-lysozyme 5. (*: p<0.05, ** p<0.001, *** p<0.0001), (Kruskal-Wallis test, Dunns post test).

In *B*. *azoricus*, the gill tissue is both a first barrier between the organism and the environment, and a symbiotic tissue. Thus, gill cells should be able, at the same time, to fight against pathogens and to allow symbiotic bacteria to penetrate and remain in the bacteriocytes. To further investigate the implication of lysozymes in symbiosis, we analysed the relative expression of the six paralogs in gills of individuals sampled from the natural population (Fig 5). It appears that Ba-lysozymes 3 and 6 have a significantly higher expression in gills compared to Ba-lysozymes 1, 4 and 5. In addition Ba-lysozyme 4 has the lowest expression in gills.

### *In situ* experiment: Relative quantification of SOX/MOX content, expression of Ba-lysozymes paralogs and antimicrobial activity in gills of *B*. *azoricus*

**Fig 6:**
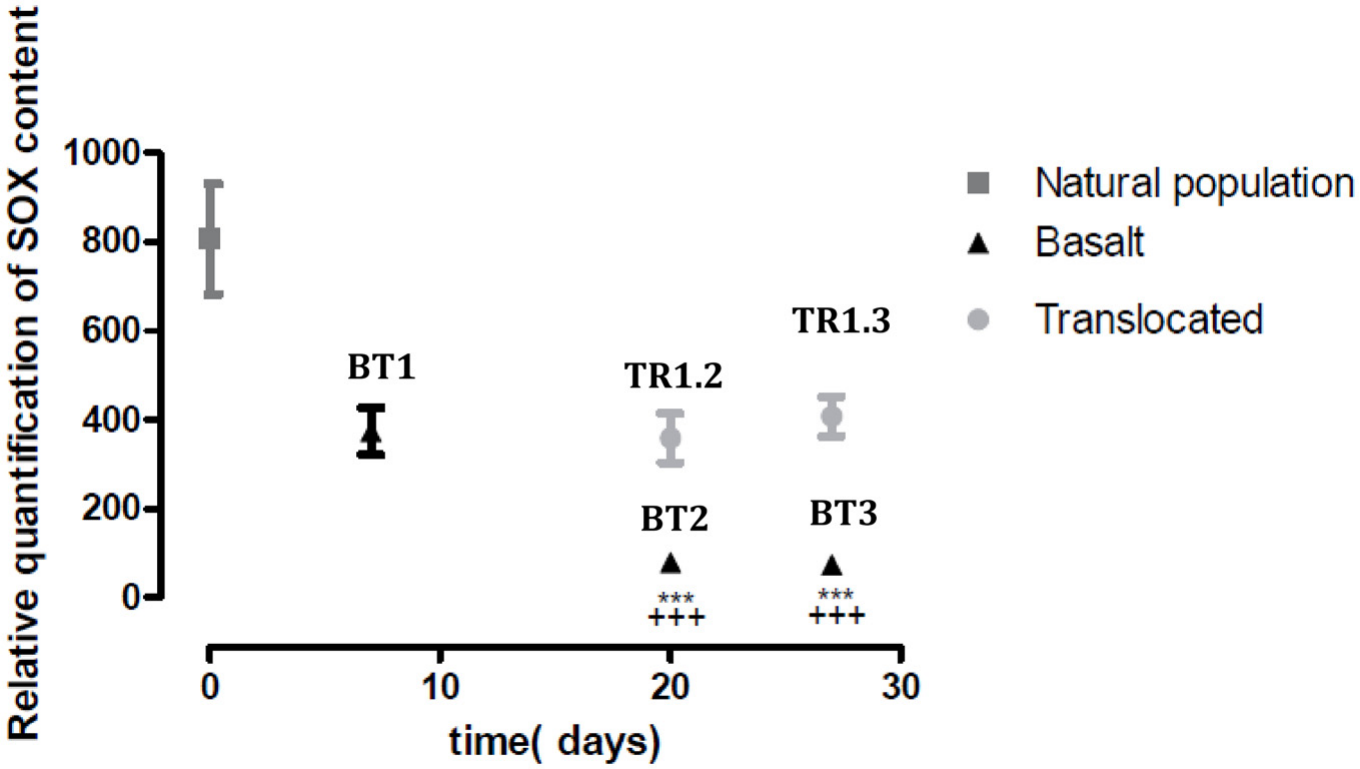
Relative quantification of SOX content in gills of *B*. *azoricus* in three conditions: Natural population (dark grey), Basalt (BT1, BT2, BT3) (black) and Translocated (grey). Relative quantification of SOX and MOX symbionts was estimated by using the comparative Ct method formula: relative quantification = 2^-ΔCt^, (ΔCt = Ct_16S_-Ct_cMDH_) with cMDH as an internal control. *: significantly different Natural population and +: significantly different from Basalt T1. (*** or +++ p<0.001), (Kruskal-Wallis test and Dunns post test).

The main goal of the experiment conducted in this study was to provoke a partial loss of symbiotic bacteria by translocating mussels in an area devoid of venting (basalt) for 6 days (BT1), 20 days (BT2) and 27 days (BT3). Then, in order to induce a reacquisition or maintenance of symbionts, we re-exposed individuals (that spend 6 days on basalt) to venting on their original location within the mussel bed for 14 and 21 days (respectively TR1.2 and TR1.3). SOX and MOX relative quantification was performed on samples from natural population, basalt (BT1, BT2, and BT3) and translocated (TR1.2, TR1.3) to assess the loss, maintenance or re-acquisition of symbionts. MOX symbiont content showed relatively very low values (below 10U) in all mussels from *in situ* population and almost no MOX was detected in experimental samples. Therefore, the MOX content has not been examined in further analysis. The variation in SOX content is presented on Fig 6. The Kruskall-Wallis test does not show significant difference in SOX content between natural population and basalt T1 individuals but a significant decrease of SOX content is observed between natural population, and BT2 and BT3. Moreover, samples from BT2 and BT3 present a significant lower SOX content than those from BT1. In contrast no significant differences were observed between samples from natural population, BT1, TR1.2 and TR1.3. These results reveal a decrease in symbiont content in a time dependent way in individuals kept on basalt, and maintenance of SOX population in gills of translocated individuals (either TR1.2 or TR1.3). Level of both SOX and MOX content observed in the Montsegur population are comparable with those previously observed in other populations from Lucky Strike [7,58] and are coherent with the general trend observed. Similar transplant experiments were performed previously in *B*. *thermophilus* in which the loss of symbiont was almost complete after only 12 days [59]. In our case, for *B*. *azoricus* at Lucky Strike, the loss of symbiont process seems to be slower and not really detectable after 6 days suggesting resilience in the symbiont degradation process in the absence of gas.

**Fig 7:**
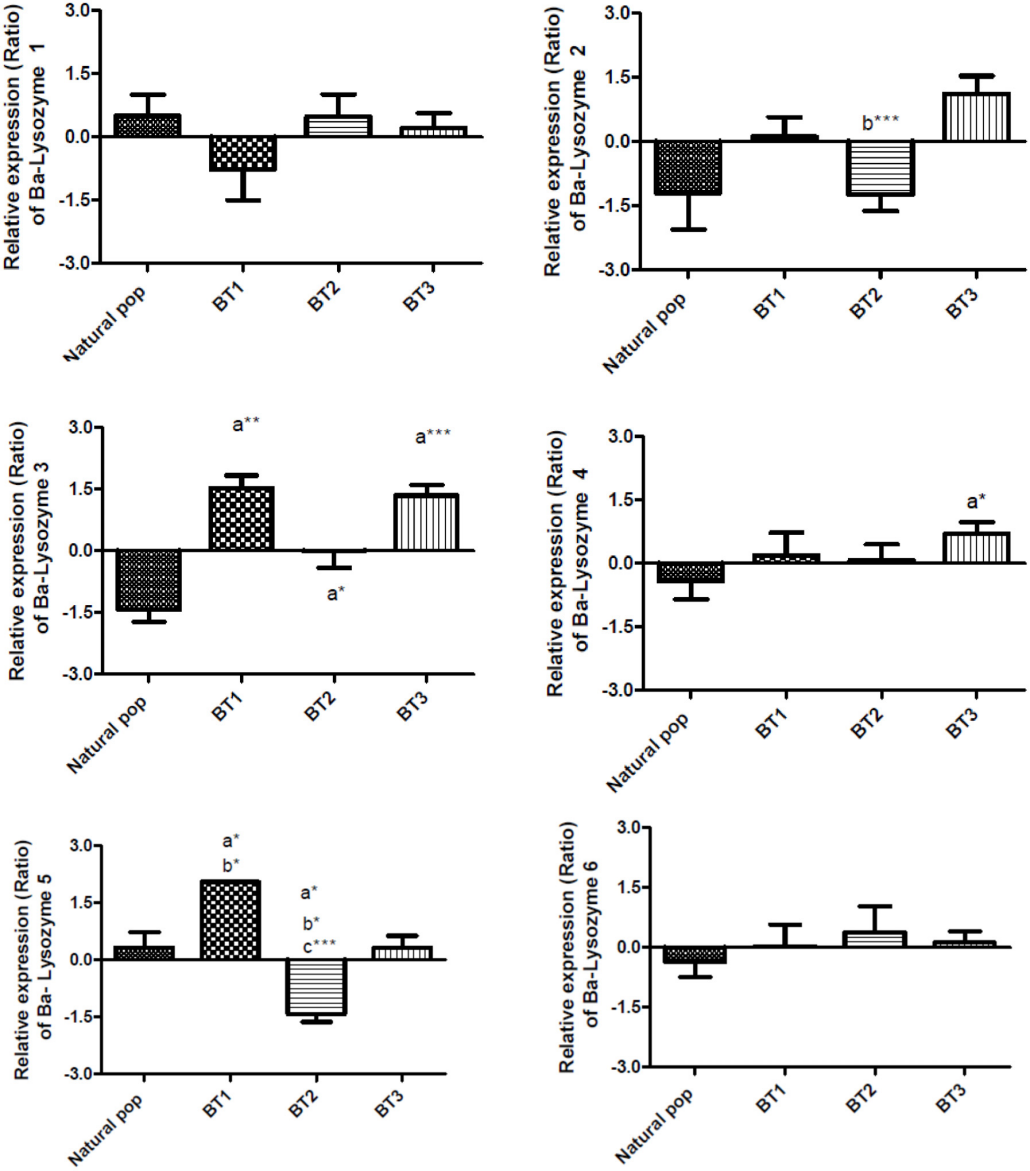
Relative expression level of lysozymes paralogs in *B*. *azoricus* gills in natural population and after symbionts loss on basalt (BT1, BT2 and BT3). Bars show the relative expression level of mRNA for each condition according to the comparative Ct method: Ratio = 2^-ΔΔCt^ (with ΔCt_geneX_ = Ct_GeneX_-Ct_RpL15_; ΔΔCt = ΔCt_geneX_-meanΔCt_geneX_) with RpL15 as an internal control. Relative expression level of mRNA (Ratio) is indicated in: Natural population (n = 31), BT1 (n = 15), BT2 (n = 24) and BT3 (n = 23). a: significantly different from natural population; b: significantly different from Basalt T3; c: significantly different from Basalt T1. (*: p<0.05, ** p<0.001, *** p<0.0001), (Kruskal-Wallis test, Dunns post test).

**Fig 8:**
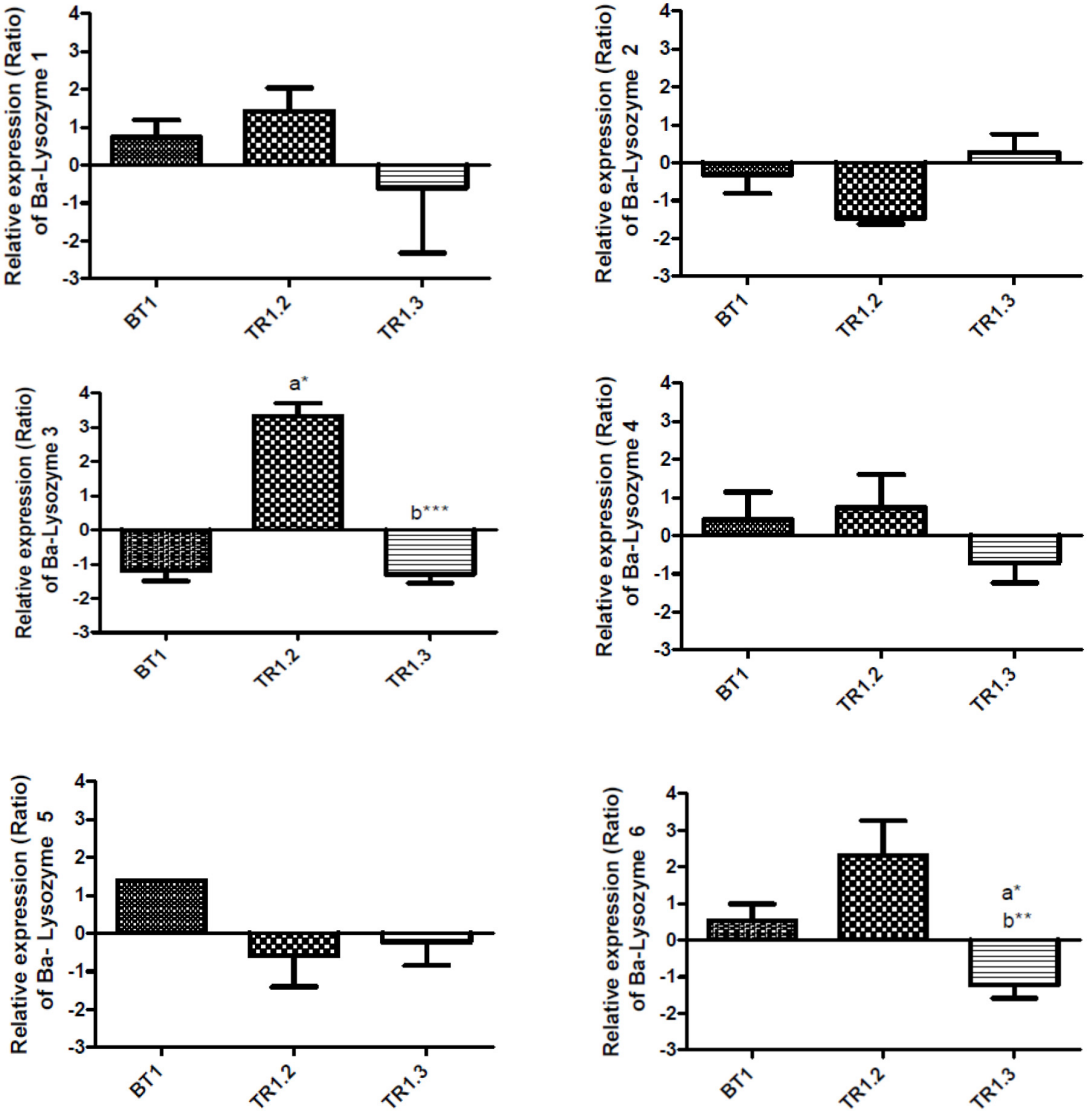
Relative expression of lysozymes paralogs in *B*. *azoricus* gills in BT1 and after a re-exposition to venting area (TR1.2 and TR1.3). Bars show the relative expression level of mRNA for each condition according to the comparative Ct method: Ratio = 2^-ΔΔCt^ (with ΔCt_geneX_ = Ct_GeneX_-Ct_RpL15_; ΔΔCt = ΔCt_geneX_-meanΔCt_geneX_) with RpL15 as an internal control. Relative expression level of mRNA (Ratio) is indicated for each condition: BT1 (n = 15), TR1.2 (n = 10) and TR1.3 (n = 11). a: Significantly different from BT1; b: significantly different from TR1.2. (*: p<0.05, ** p<0.001, *** p<0.0001), (Kruskal-Wallis test, Dunns post test).

The relative expression of the 6 Ba-lysozyme paralogs was measured by qPCR in the symbiotic organ (gill) in all experimental samples. In order to observe the impact of the loss of symbionts on the expression of lysozymes paralogs, we first compared samples from natural population (LS, Montsegur) with those with a lower symbiotic content (BT1, BT2 and BT3) (Fig 7). Secondly, we compared lysozymes relative expression between individuals with a weak loss of symbionts (BT1) and individuals issued from the re-exposition to venting area (TR1.2 and TR1.3) (Fig 8).

The first observation is a general up-regulation trend in BT1 condition only significant for Ba-lysozyme 3 and 5 (Fig 7). After a longer period on basalt (BT2), Ba-lysozyme 3 and 5 are significantly down-regulated compared to natural population while the expression of paralogs 4 is not modified. For BT3, Ba-lysozyme 5 seems to return to a level of expression similar to the one observed in natural population whereas Ba-lysozyme 3 and 4 are up-regulated. Expression of Ba-lysozyme 2 on basalt (BT3) in comparison with natural population is not significantly different due to an important variability of this paralog expression in natural population but it is significantly up-regulated in BT3 compared to BT2. The downward trend of symbiotic charge observed after 6 days on basalt (BT1) seems to induce an increase of Ba-lysozyme 3 and 5 expression. This up-regulation can be due to either a lysis of useless bacteria or a stress response to a changing environment. After 20 days (BT2), with the significant decrease of symbiotic charge, we observed a significant up-regulation of Ba-lysozyme 3 and a significant down-regulation of Ba-lysozyme 5 in comparison with natural population. After 27 days on basalt (BT3), while the symbiotic charge remains low, Ba-lysozyme 3 and 4 are up-regulated in comparison with natural population. Among these four paralogs, Ba-lysozyme 3 has an interesting pattern as it is up-regulated in the three basalt conditions. Finally, the impact of the loss of symbiont is different in these six paralogs, two are not impacted (1 and 6), three have their expression increased after 27 days on basalt (BT3) compared to natural population (paralogs 2, 3 and 4), and one return to an expression similar to the natural population (paralog 5).

In the second analysis, individuals from the translocation experiment (TR1.2 and TR1.3) were compared with those from BT1 (Fig 8). Two paralogs were differentially expressed, in this comparison (Fig 8). The translocation induces an up-regulation of Ba-lysozyme 3, 6, only significant for 3 (TR1.2). It is followed by a significant down regulation of Ba-lysozyme 3 and 6 compared to TR1.2. Finally in TR1.3, expression level of Ba-lysozyme 3 returns to the level of BT1. These results suggest that Ba-lysozyme 3 and 6 could be implicated in the loss of symbiont but not in the maintenance of symbiotic content in gills.

**Fig 9:**
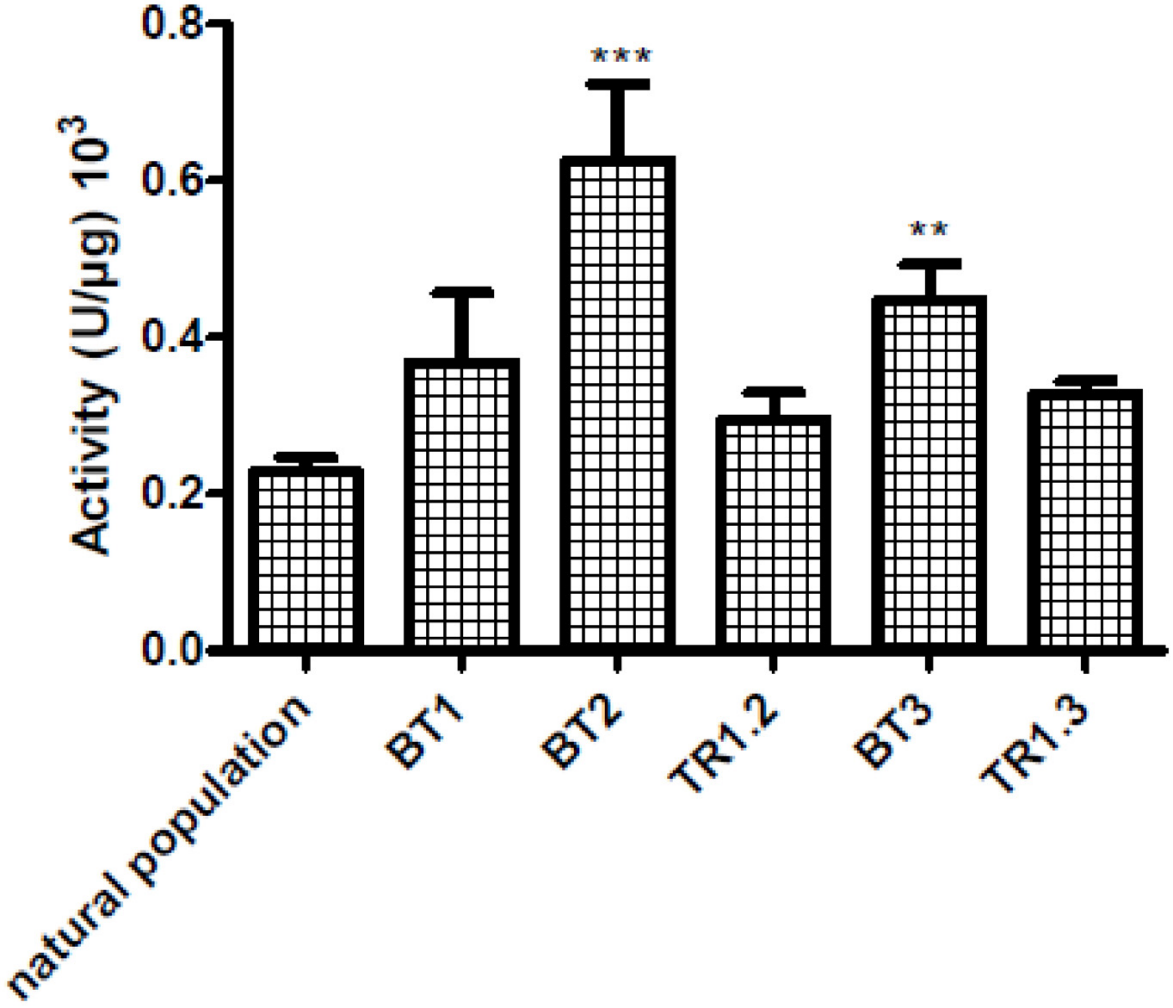
Global lysozyme activity in *B*.*azoricus* gills in different conditions: Natural population (n = 81), BT1 (n = 12), BT2 (n = 21), BT3 (n = 15), TR1.2 (n = 10), and TR1.3 (n = 11). Lysozyme activity is expressed by units of μg of proteins. *: significantly different from natural population. (*: p<0.05, ** p<0.001, *** p<0.0001), (Kruskal-Wallis test, Dunns post test).

Global lysozyme activity was measured in gills from all natural and experimental samples (natural population, BT1, BT2, BT3 and TR1.2 and 1.3) (Fig 9). On the one hand, lysozyme anti-microbial activity increased significantly after 20 and 27 days in samples from BT2 and BT3 with the highest activity in BT2 individuals. The global induction of anti-microbial activity appears to be linked with the loss of symbiont and also with the up-regulation of the three Ba-lysozymes paralogs mentioned earlier, *i*.*e*. Ba-Lysozyme 2, 3 and 4. On the other hand, no differences of anti-microbial activity were found between BT1 and translocated individuals.

## Concluding Remarks

In this study we have demonstrated the presence of six paralogs of i-type lysozymes in the deep sea hydrothermal vent mussel *Bathymodiolus azoricus*. Some of these paralogs were found differentially regulated in different tissues and under different symbiotic state. Ba-lysozyme 6 expression seems specific to the mantle compared to other tissue but its expression in gills is also very high. Ba-lysozyme 3 is an interesting candidate for our study as it is up-regulated in gills compared to other tissues and it has one of the strongest expressions in this tissue compared to other paralogs. Moreover, the loss of symbiont induces an up-regulation of this paralog suggesting its implication in a possible digestion of symbionts. The loss of symbionts induces an up-regulation of two others paralogs (2 and 4) which had a low expression in gills of natural population. In addition, within this tissue, the global anti-microbial activity increased with the loss of symbiont.

Lysozymes are thought to play a key role in symbiosis in invertebrates. In the symbiotic association between the pea aphid *Acyrthosiphon pisum* and *Buchnera*, Nakabachi and collaborators observed an expression level of lysozyme 156 times higher in bacteriocytes than in the whole body [35]. More recently, Nishikori and co-authors observed a drastic decrease in *Buchnera* density during stage 1 associated with an increase of the expression of *lysozyme i-1* and an increase of *Buchnera* degradation [60]. In addition, expression of c-type lysozyme, measured during the pre-molt stages of the insect *Riptortus* increased when symbiont population decreased. This expression was found identical in aposymbiotic individuals [61] suggesting the implication of lysozymes in symbiont regulation. Similarly, Anselme as well as Futahashi and their co-authors observed an increase of lysozyme expression in aposymbiotic individuals (respectively Weevil and Stinkbug) [62,63].

Although, we cannot completely exclude that part of the lysosomal activity in the gill tissue could correspond to a contribution of circulating hemocytes (potentially responding to pathogenic bacteria as described in clams, [64]), our results strongly support the role of lysozymes in intracellular digestion of symbionts. The digestion could be a way for the host to regulate and eliminate excess bacteria (either in too large numbers or without access to reduced chemicals) but also to have access to a source of nutrient after a long-term starvation [20]. Indeed, in another chemoautotrophic endosymbiosis, cytochemical experiment showed a degradation of bacterial cell wall in gills after two weeks of starvation. Authors made the hypothesis that the host uses symbionts as nutrient to survive a hunger crisis [65]. All together our results suggest an implication of Ba-lysozymes in the digestion of symbionts in *Bathymodiolus azoricus* bacteriocytes, and therefore a potential role in symbiosis. To go further, it would be of particular interest to make a link between the structure and function (immunity, digestion or involvement in symbiosis) of lysozymes by purifying each Ba-lysozymes paralogs and measuring their antimicrobial activity. Moreover ultrastructural experiments and immunogold-labelling on gills of individuals from *in situ* experiments might provide evidences to confirm or not the role of lysozymes in *B*. *azoricus* symbiosis.

## Supporting Information

S1 File Nucleotide sequences of Ba-lysozyme 1 to 6 (Figure A) and Ribbon representation of the 3D model of Ba-lyzozyme 4 (Figure B). (DOCX)
