# Supplementary material for: Multiple I-Type Lysozymes in the Hydrothermal Vent Mussel Bathymodiolus azoricus and Their Role in Symbiotic Plasticity
Source: PLoS One. 2016 Feb 16;11(2):e0148988. doi: 10.1371/journal.pone.0148988 (PMC4755537; doi:10.1371/journal.pone.0148988)
Supplement: S1 File — (DOCX) [file pone.0148988.s001.docx]

**Ba-lysozyme 1**

**atg**tctcctcgagtttatttggtgttacttttgccttttatgtcccaagttcaaccaaat

M S P R V Y L V L L L P F M S Q V Q P N

gacttagtttccccaaaatgtatgagctgtatctgtcaggttgaatcgcactgcctaccc

D L V S P K C M S C I C Q V E S H C L P

ataggatgtcgtatggatgttggttcgttgtcctgtggacctttccaaattaaaaagggt

I G C R M D V G S L S C G P F Q I K K G

tattggacagattgtggtagtccaggcggagattatgaaacatgcacaagagactacaca

Y W T D C G S P G G D Y E T C T R D Y T

tgttcttacaactgtgttcaaagatacatggcaagatatataaaattcagcggatgtctc

C S Y N C V Q R Y M A R Y I K F S G C L

aagaactgtgaaagctatgccaggatccataacggaggaccacgagggtgtacaaatcca

K N C E S Y A R I H N G G P R G C T N P

aatacaatgggctactggaagaaaatggaatcaaagggctattatgaaacatgcactaaa

N T M G Y W K K M E S K G Y Y E T C T K

gacttcagatgttcttacaactgtgttcaaagatacatggcaagatatacaaaattcagc

D F R C S Y N C V Q R Y M A R Y T K F S

ggatgtctcaagaactgtgaaagctatgccaggatccataacggaggaccacgagggtgt

G C L K N C E S Y A R I H N G G P R G C

acaaggccaaacacaatgggctactggaagaaaatggaatcaaagggctgtactccatac

T R P N T M G Y W K K M E S K G C T P Y

agc**taa**

S -

**Ba-lysozyme 2**

**atg**gtgtcatctgtgacgtcatcgcttatactgttaccgtttctttctgtcgctctacgt

M V S S V T S S L I L L P F L S V A L R

gaatcagaatatgttaaaggaatttctgatcaatgcttaaagtgtatatgtgatgtcgaa

E S E Y V K G I S D Q C L K C I C D V E

acaaattgcgatcataacacagggtgtgctgatgatgaaggtacaccctcatgtggccca

T N C D H N T G C A D D E G T P S C G P

tttcaaataaaggaagtttattggattgactgcggcagaccaggaaaaagctatgagcaa

F Q I K E V Y W I D C G R P G K S Y E Q

tgtagcaaagactacaactgttcgagaggttgtgtaaaggcctacatgaaacgctatggt

C S K D Y N C S R G C V K A Y M K R Y G

aataaggtgtgtgaggcaaactgcgagggatttgctcgtatgcacaatggaggtccaaga

N K V C E A N C E G F A R M H N G G P R

ggatgcttgaaatatgaaacagaaaaatattggagtaaaatgatgaaagcaggctgtggt

G C L K Y E T E K Y W S K M M K A G C G

ggaataaaaggc**tag**

G I K G -

**Ba-lysozyme 3**

**atg**tttgttaaaatgtcaccactagtgtatttggtattactcctgccttttatgtcccag

M F V K M S P L V Y L V L L L P F M S Q

gtttgtagtaccttggacattgttgaaaattttgaaatagagtttgaacagattgatgta

V C S T L D I V E N F E I E F E Q I D V

gaagttcaaccaaatgacttagtttccccaaaatgtatgagctgtatctgtcaggttgaa

E V Q P N D L V S P K C M S C I C Q V E

tcgcactgcctacccataggatgtcgtatggatgttggttcgttgtcctgtggacctttc

S H C L P I G C R M D V G S L S C G P F

caaattaaaaagggttattggacagattgtggtagtccaggcggagattatgaaacatgc

Q I K K G Y W T D C G S P G G D Y E T C

acaagagactacacatgttcttacaactgtgttcaaagatacatggcaagatatataaaa

T R D Y T C S Y N C V Q R Y M A R Y I K

ttcagcggatgtctcaagaactgtgaaagctatgccaggatccataacggaggaccacga

F S G C L K N C E S Y A R I H N G G P R

gggtgtacaaatccaaatacaatgggctactggaagaaaatggaatcaaagggctgtact

G C T N P N T M G Y W K K M E S K G C T

ccatacagc**taa**

P Y S -

**Ba-lysozyme 4**

**atg**aaaaagatgatgattattgcaggaattgttgccttgctgtgctttacatattcggca

M K K M M I I A G I V A L L C F T Y S A

gagggtgctgtatctggtaactgcttggattgtatttggcaggttgaatcaggaaaagca

E G A V S G N C L D C I W Q V E S G K A

agccatctgggatgtagcatggatgttggttcaagagcttgtggcccgtatcaaatacat

S H L G C S M D V G S R A C G P Y Q I H

cacgattattacaaagattgcacccatgatgatgacgcgagtgtgacagagtgggaagct

H D Y Y K D C T H D D D A S V T E W E A

tgcacacaggaggccgactgttctcgaacatgcgttcaagactatatgaaacgttacgga

C T Q E A D C S R T C V Q D Y M K R Y G

ggaaagaaatgtccaaacgattgtgaaggctacgctagaatgcacaacggtggacctgat

G K K C P N D C E G Y A R M H N G G P D

gggtgtaataaatctcaaacagacgtttactggaacaaaattaaccgtgctggctgttct

G C N K S Q T D V Y W N K I N R A G C S

agtcacagt**taa**

S H S -

**Ba-lysozyme 5**

**atg**tcgaagttatctgttcttttggtcgttgttttcatggttctgactacaaaagtcaat

M S K L S V L L V V V F M V L T T K V N

tttggattttcagcaggaatctctgacaagtgtcttcagtgtatctgtgacgcggagacg

F G F S A G I S D K C L Q C I C D A E T

aattgtgatgcaactctacaatgcgaagacgatggtggtaccccctcttgtggtcccttc

N C D A T L Q C E D D G G T P S C G P F

aagatcaaaaaagtttattggctagactgtggcaaaccaggaaacagttttgaggagtgt

K I K K V Y W L D C G K P G N S F E E C

gctaaggactatgaatgttccactga

A K D Y E C S T

**Ba-lysozyme 6**

ggtccattacggccggggaacagattgatgtagaagttcaaccaaa**tga**cttagtttcccc

caaaaatgt**atg**agctgtatctgtcaggttgaatcgcactgccaccccataggatgtcgt

M S C I C Q V E S H C H P I G C R

atggatgttggttcgttgtcctgtggacctttccaaattaaaaagggttattggacagat

M D V G S L S C G P F Q I K K G Y W T D

tgtggtagtccaggcggagattatgaaacatgcacaagagactacacatgttcttacaac

C G S P G G D Y E T C T R D Y T C S Y N

tgtgttcaaagatacatggcaagatatataaaattcagcggatgtctcaagaactgtgaa

C V Q R Y M A R Y I K F S G C L K N C E

agctatgccaggatccataacggaggaccacgagggtgtacaaatccaaatacaatgggc

S Y A R I H N G G P R G C T N P N T M G

tactggaagaaaatggaatcaaagggctgtactccatacagc**taa**

Y W K K M E S K G C T P Y S -

Figure A: Nucleotide sequences of Ba-lysozyme 1 to 6

The start and stop codons are indicated in bold.


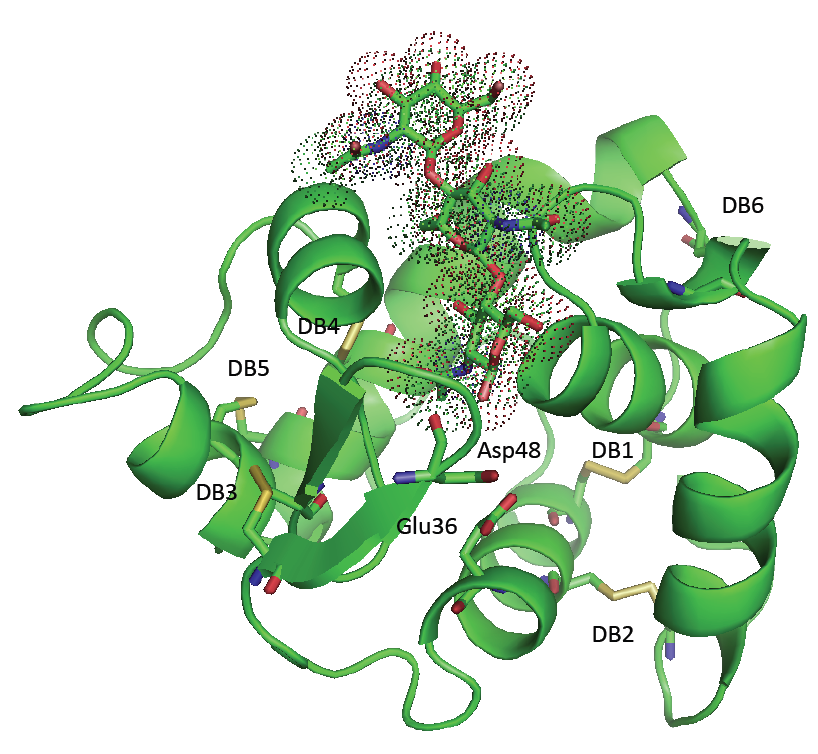


Figure B : Ribbon representation of the 3D model of Ba-lyzozyme 4. This model was obtained by homology modeling (Modeller 9v8, Template lysozyme from *Meretrix lusoria*, Code pdb 3AB6). Three NAG units are bound (dots). The two catalytic residues, Asp 48 and Glu36, and the six disulfide bridges (DB 1 to 6) are presented.
